# Effect of *Ishige okamurae* Extract on Osteoclastogenesis In Vitro and In Vivo

**DOI:** 10.3390/md22030137

**Published:** 2024-03-20

**Authors:** Su-Hyeon Cho, Hyun-Soo Kim, Juhee Ahn, Bomi Ryu, Jun-Geon Jea, Kyubin Lee, Kyunghwan Kim, Ginnae Ahn, WonWoo Lee, Kyung-Min Choi, Kil-Nam Kim

**Affiliations:** 1 Gwangju Center, Korea Basic Science Institute (KBSI), Gwangju 61751, Republic of Korea; chosh93@kbsi.re.kr; 2 Department of Seafood Science and Technology, The Institute of Marine Industry, Gyeongsang National University, Tongyeong 53064, Republic of Korea; gustn783@naver.com; 3 Department of Medical Biomaterials Engineering, Kangwon National University, Chuncheon 24341, Republic of Korea; juheeahn@kangwon.ac.kr; 4 Department of Food Science and Nutrition, Pukyung National University, Busan 48513, Republic of Korea; bmryu@pknu.ac.kr; 5 Department of Marine Life Sciences, Jeju National University, Jeju 63243, Republic of Korea; wpwnsrjs@gmail.com; 6 Department of Biological Sciences and Biotechnology, Chungbuk National University, Chungbuk 28644, Republic of Korea; kbl816@chungbuk.ac.kr (K.L.); kyungkim@chungbuk.ac.kr (K.K.); 7 Department of Marine Bio-Food Sciences, Chonnam National University, Yeosu 59626, Republic of Korea; gnahn@chonnam.ac.kr; 8 Honam National Institute of Biological Resources (HNIBR), Mokpo 58762, Republic of Korea; 21cow@hnibr.re.kr (W.L.); kyungmc0111@hnibr.re.kr (K.-M.C.); 9 Department of Bio-Analysis Science, University of Science & Technology, Daejeon 34113, Republic of Korea

**Keywords:** *Ishige okamurae*, osteoclasts, NF-κB, MAPK, RAW 264.7 cells, zebrafish larvae

## Abstract

We demonstrated the effect of *Ishige okamurae* extract (IOE) on the receptor activator of nuclear factor-κB ligand (RANKL)-promoted osteoclastogenesis in RAW 264.7 cells and confirmed that IOE inhibited RANKL-induced tartrate-resistant acid phosphatase (TRAP) activity and osteoclast differentiation. IOE inhibited protein expression of TRAP, metallopeptidase-9 (MMP-9), the calcitonin receptor (CTR), and cathepsin K (CTK). IOE treatment suppressed the expression of activated T cell cytoplasmic 1 and activator protein-1, thus controlling the expression of osteoclast-related factors. Moreover, IOE significantly reduced RANKL-phosphorylated extracellular signal-regulated kinase (ERK) and c-Jun N-terminal kinase (JNK). It also reduced the RANKL-induced phosphorylation of NF-κB and nuclear translocation of p65. IOE inhibited Dex-induced bone loss and osteoclast-related gene expression in zebrafish larvae. HPLC analysis shows that IOE consists of 3.13% and 3.42% DPHC and IPA, respectively. Our results show that IOE has inhibitory effects on osteoclastogenesis in vitro and in vivo and is a potential therapeutic for osteoporosis.

## 1. Introduction

Bone is a rigid tissue that plays many roles such as protecting various organs, generating red and white blood cells, storing minerals, and ensuring mobility [1]. Bone homeostasis is controlled by mechanisms that maintain the balance between bone resorption and bone formation [2]. Normal bone continuously undergoes remodeling under the control of osteoclasts and osteoblasts [3,4]. However, impaired regulation of bone resorption and formation results in various metabolic bone diseases such as osteoporosis [2,5].

Osteoclasts, which are multinucleated giant cells, differentiate from osteoclast precursor cells originating from hematopoietic stem cell mononuclear/macrophage lineage cells in the bone marrow [3,6]. Excessive osteoclastic activity causes extensive bone resorption, which often manifests in bone diseases such as osteoporosis [3]. Osteoporosis is the most common metabolic skeletal bone disorder and is characterized by bone loss, microarchitectural deterioration of bone tissue, and an increased risk of bone fracture [2,7]. Osteoclast differentiation is initiated by the receptor activator of nuclear factor-κB ligand (RANKL). The binding of RANKL and its receptor RANK onto osteoclast precursor cell surfaces initiates the recruitment of tumor necrosis factor receptor-associated factor 6 (TRAF6) [8,9]. This process stimulates a cascade of downstream pathways, such as nuclear factor kappa B (NF-κB) and mitogen-activated protein kinases (MAPKs) [10]. Moreover, these signaling pathways activate the nuclear factor of activated T cell cytoplasmic 1 (NFATc1), which is an important transcriptional factor for osteoclast differentiation [10,11]. Activated NFATc1 promotes the expression of osteoclastogenesis-related proteins, including tartrate-resistant acid phosphatase (TRAP), the calcitonin receptor (CTR), cathepsin K (CTK), and metallopeptidase-9 (MMP-9) [8]. Therefore, suppressing excessive osteoclast activity is essential for treating osteoporosis.

Bisphosphates have been widely used as first-line therapy to manage osteoporotic fracture risks and function by inhibiting the bone resorption function of osteoclasts [12,13]. Although they are generally well tolerated when administered properly, potential adverse effects, such as severe muscle skeletal pain, osteonecrosis of the jaw, unusual fractures in the femur (tight bone) and the shaft (diaphysis or sub-trochanteric region) of the bone, and the suppression of bone turnover, have been reported [14,15]. Natural products have been demonstrated to exhibit similar therapeutic biological activity but with higher levels of safety, and fewer side effects [16,17]. Therefore, it serves as an important source of alternative agents for osteoporosis treatment.

Marine algae contain various bioactive substances, such as polyphenols, proteins, polysaccharides, and lipids, and exhibit pharmaceutical, nutraceutical, and biomedical potential in functional foods [18,19,20]. Marine algae have many bioactive compounds with diverse biological activities, including anti-inflammatory, anti-melanogenesis, antioxidant, and antimicrobial activities, which have been used in functional foods and biomedical sources [20,21,22]. *Ishige okamurae*, a brown alga, is a member of the Ishigeaceae family and is an edible seaweed inhabiting temperate coastal zone areas in Korea, Japan, and China [23,24]. It is widely used as a food ingredient and in traditional medicine [25]. Recent studies have reported a number of biological activities exhibited by *I. okamurae* extract (IOE), including anti-inflammatory, anti-diabetic, and inhibitory effects on HIV [25,26,27]. *I. okamurae* contains many bioactive compounds such as fucoxanthin, phloroglucinol, diphlorethohydroxycarmalol (DPHC), and ishophloroglucin A (IPA) [28,29,30,31]. DPHC has been demonstrated to inhibit osteoclastogenesis by blocking the activation of the NF-κB signaling pathway in vitro [30]. Moreover, IPA has an effective inhibitory activity against osteoclastogenesis in vitro [32]. However, the effect of IOE on osteoclastogenesis inhibition has not yet been demonstrated. In this study, we demonstrated the effects of IOE on RANKL-induced osteoclastogenesis on RAW 264.7 cells and dexamethasone-induced zebrafish larvae.

## 2. Results

### 2.1. Effect of IOE on TRAP Activity in RANKL-Stimulated RAW 264.7 Cells

**Figure 1:**
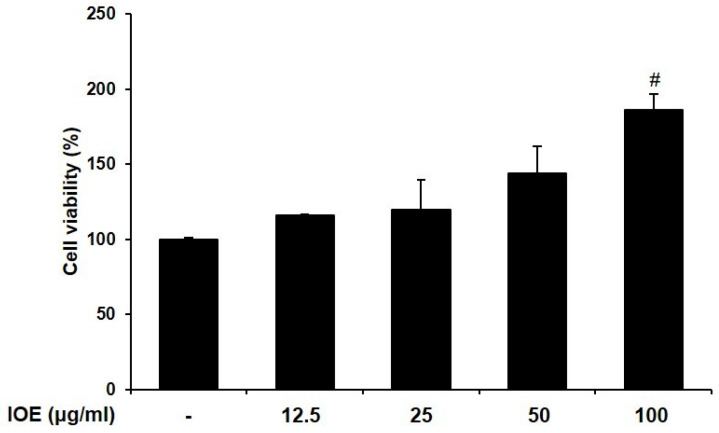
Effect of IOE on cell viability in RAW 264.7 cells. The cells were treated with 12.5, 25, 50, and 100 μg/mL of IOE for 5 days. Cell viability was assessed with MTT assay. All results are expressed as mean ± standard deviation (SD) from more than three individual experiments; # *p* < 0.05 compared with the control group.

**Figure 2:**
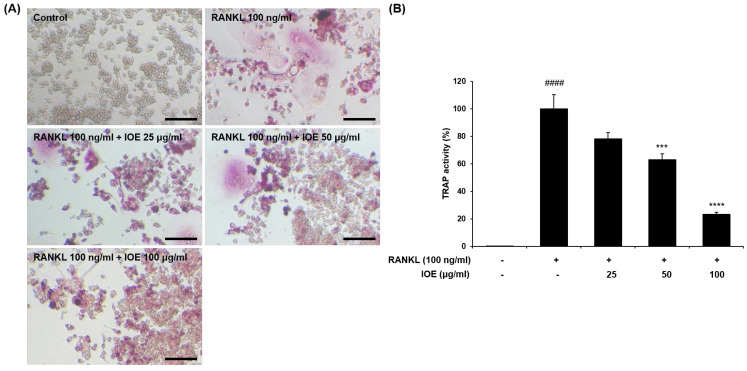
Effect of IOE on TRAP activity in RANKL-induced RAW 264.7 cells. Cells were pre-treated with 25, 50, and 100 μg/mL of IOE for 2 h and then treated with 100 ng/mL of RANKL for 5 days. TRAP activity was measured with TRAP staining. (**A**) Representative images of TRAP staining (scale bar: 100 μm). IOE treatment inhibited RANKL-induced TRAP activity and osteoclast differentiation. (**B**) Counted TRAP positive cells. All results are expressed as mean ± standard deviation (SD) from more than three individual experiments; #### *p* < 0.0001 compared with the control group. *** *p* < 0.001, and **** *p* < 0.0001 compared with the RANKL-stimulated group.

We first confirmed the viability of RAW 264.7 cells treated with IOE and found that this preparation was not cytotoxic at concentrations up to 100 μg/mL (Figure 1). These non-cytotoxic concentrations were used to demonstrate the effect of IOE on RAW 264.7 cell osteoclastogenesis. Next, to investigate the effect of IOE on RANKL-differentiated osteoclasts in cells, we stained the cells with TRAP, an osteoclast-related factor (Figure 2). As shown in Figure 2, RANKL promoted osteoclast differentiation and TRAP activity compared to that seen in the control group, but IOE treatment significantly reduced osteoclast differentiation and TRAP activity in a dose-dependent manner.

### 2.2. Effect of IOE on the Expression of Osteoclast Differentiation-Related Factors in RANKL-Stimulated RAW 264.7 Cells

**Figure 3:**
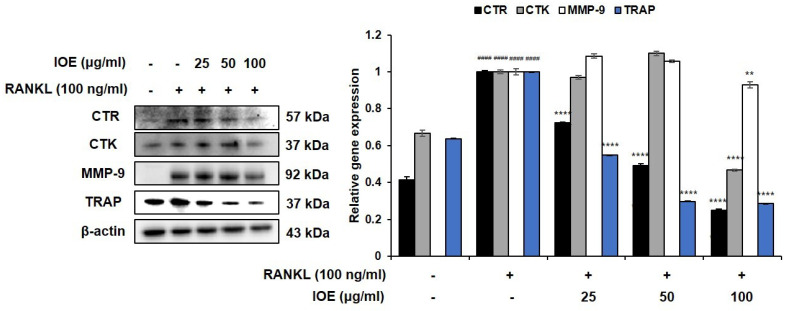
Effect of IOE on osteoclast-related factors in RANKL-induced RAW 264.7 cells. Cells were pre-treated with 25, 50, and 100 μg/mL for 2 h and then treated with 100 ng/mL of RANKL for 5 days. Protein expression of osteoclast-related factors CTR, CTK, MMP-9, and TRAP. All results are expressed as mean ± standard deviation (SD) from more than three individual experiments; #### *p* < 0.0001 compared with the control group. ** *p* < 0.01, and **** *p* < 0.0001 compared with the RANKL-stimulated group.

We next analyzed the expression levels of osteoclast differentiation-related proteins in RANKL-induced RAW 264.7 cells. As shown in Figure 3, RANKL promoted CTR, CTK, MMP-9, and TRAP expression compared with that seen in the control group. However, IOE markedly suppressed CTR and TRAP expression in a dose-dependent manner. CTK and MMP’s expression levels were significantly inhibited in the 100 μg/mL IOE-treated group. These results show that IOE reduces RANKL-induced osteoclast differentiation by downregulating the osteoclast differentiation-related mediators CTR, CTK, MMP-9, and TRAP in RAW 264.7 cells.

### 2.3. Effect of IOE on the Expression of Osteoclast-Related Transcriptional Factors in RANKL-Stimulated RAW 264.7 Cells

**Figure 4:**
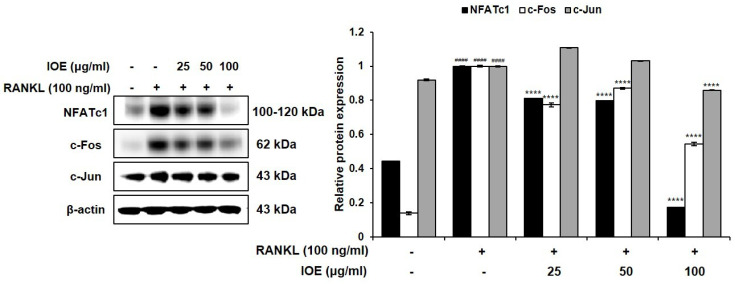
Effect of IOE on expression of osteoclast-related transcription factors in RANKL-induced RAW 264.7 cells. Cells were pre-treated with 25, 50, and 100 μg/mL of IOE for 2 h and then treated with 100 ng/mL of RANKL for 9 h. Protein expressions were evaluated using Western blotting analysis. (All results are expressed as mean ± standard deviation (SD) from more than three individual experiments; #### *p* < 0.0001 compared with the control group. **** *p* < 0.0001 compared with the RANKL-stimulated group.

We investigated the effects of IOE on the expression of the factors associated with osteoclastogenesis in RANKL-stimulated RAW 264.7 cells using Western blotting analysis. As shown in Figure 4, RANKL promoted the expression NFATc1, c-Fos, and c-Jun in RAW 264.7 cells. However, IOE treatment significantly inhibited the RANKL-induced expression of these factors in a dose-dependent manner.

### 2.4. Effect of IOE on ERK, JNK, and NF-κB Phosphorylation in RANKL-Stimulated RAW 264.7 Cells

**Figure 5:**
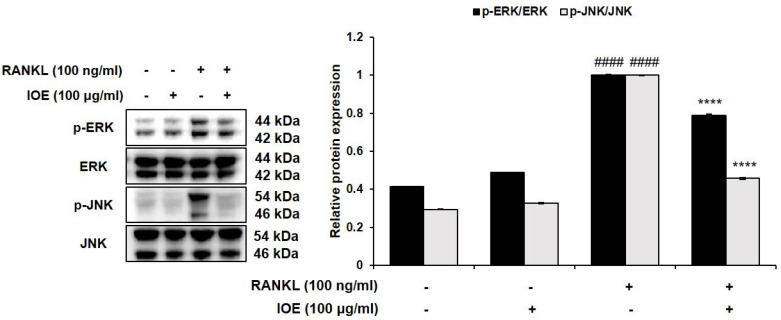
Effect of IOE on activation of ERK and JNK in RANKL-induced RAW 264.7 cells. Cells were pre-treated with 100 μg/mL of IOE for 2 h and then treated with 100 ng/mL of RANKL for 20 min. Protein levels were determined using Western blotting analysis. All results are expressed as mean ± standard deviation (SD) from more than three individual experiments; #### *p* < 0.0001 compared with the control group. **** *p* < 0.0001 compared with the RANKL-stimulated group.

**Figure 6:**
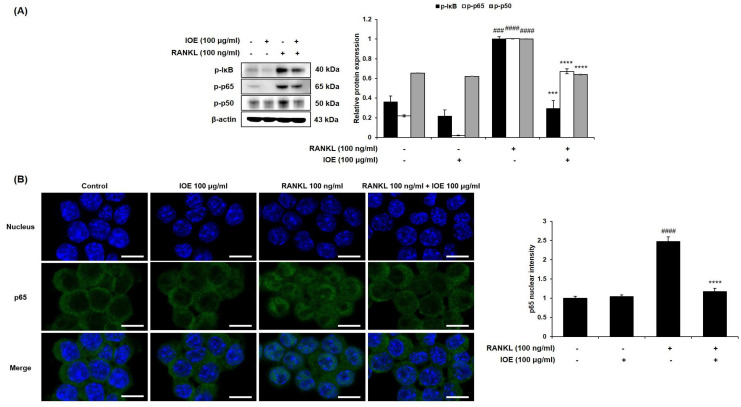
Effect of IOE on activation of NF-κB in RANKL-induced RAW 264.7 cells. Cells were pre-treated with 100 μg/mL of IOE for 2 h and then treated with 100 ng/mL of RANKL for 5 min. (**A**) Protein levels were determined using Western blotting analysis. (**B**) The nuclear translocation of p65 was observed by LSM700 Zeiss confocal laser scanning microscope (scale bar: 10 μm). Quantification of p65 nuclear translocation was performed using Image J program. All results are expressed as mean ± standard deviation (SD) from more than three individual experiments; ### *p* < 0.001, and #### *p* < 0.0001 compared with the control group. *** *p* < 0.001, and **** *p* < 0.0001 compared with the RANKL-stimulated group.

To demonstrate the role of ERK, JNK, and NF-κB activation in the IOE-induced inhibition of osteoclastogenesis, the phosphorylation of the ERK, JNK, and NF-κB signaling pathways was investigated using Western blotting analysis. Protein levels of phosphor-ERK and phospho-JNK were normalized to that of total ERK or JNK levels. As shown in Figure 5 and Figure 6, the levels of RANKL-phosphorylated ERK, JNK (Figure 5), IκB, p50, and p65 (Figure 6A) were increased compared to those in the control group. However, treatment with 100 μg/mL IOE significantly suppressed the expression of RANKL-phosphorylated ERK, JNK, and NF-κB. RANKL also induced the translocation of p65 from the cytosol to the nucleus. However, IOE inhibited the RANKL-induced nuclear translocation of p65 (Figure 6B).

### 2.5. Effect of IOE on Bone Mineralization in Dexamethasone-Induced Osteoporosis in Zebrafish Larvae

**Figure 7:**
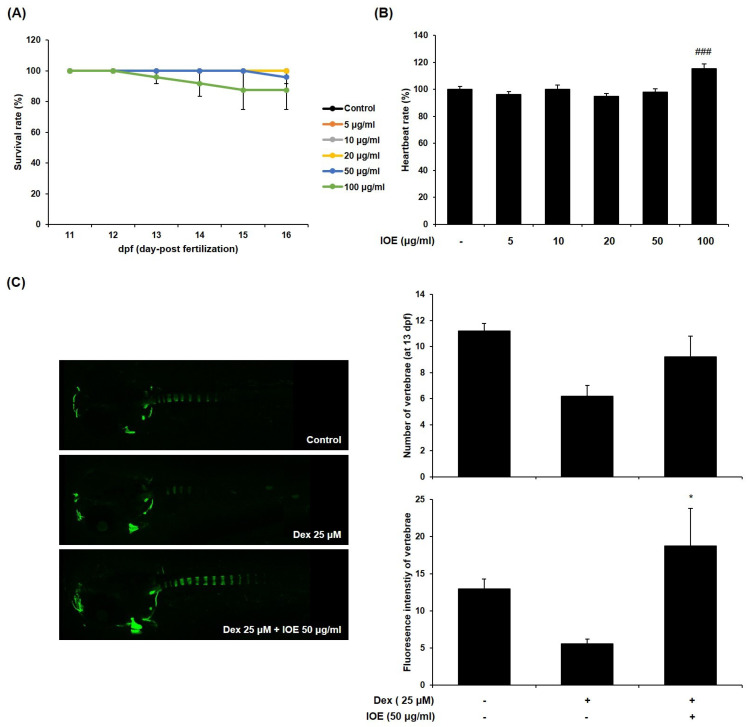
Effect of IOE on (**A**) survival rate, (**B**) heartbeat rate, and (**C**) bone mineralization in dexamethasone (Dex)-stimulated zebrafish larvae. (**A**,**B**) Zebrafish larvae (10 dpf) were treated with various concentrations of IOE (5, 10, 20, 50, and 100 μg/mL) for 6 day. (**C**) Zebrafish larvae (10 dpf) were treated with 50 μg/mL of IOE and 25 μM of dexamethasone for 3 days. Bone mineralization was evaluated using calcein staining and visualized by LSM700 Zeiss confocal laser scanning microscope. The fluorescence intensity was quantified using ImageJ 1.53t software (Java 1.8.0_345). All results are expressed as mean ± standard deviation (SD) from more than three individual experiments; ### *p* < 0.001 compared with the control group. * *p* < 0.05 with the Dex-stimulated group.

**Figure 8:**
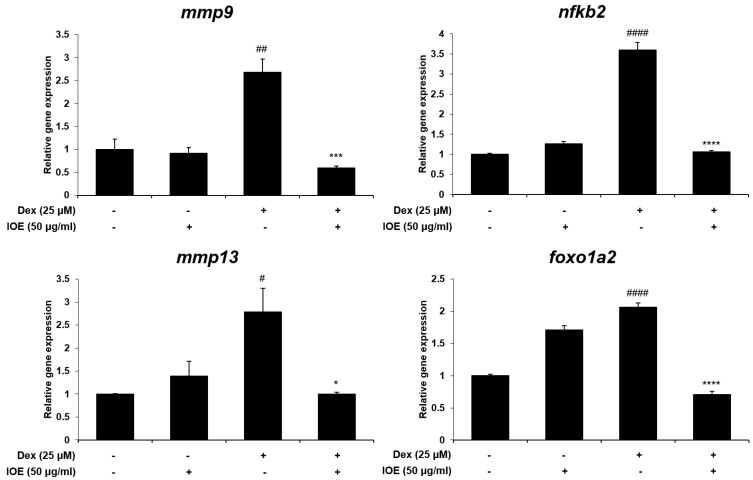
Effect of IOE on osteoclast-related gene expressions in dexamethasone-stimulated zebrafish larvae. Zebrafish larvae (10 dpf) were treated with 50 μg/mL of IOE and 25 μM of dexamethasone for 3 days. mRNA expression was evaluated using RT-qPCR analysis. All results are expressed as mean ± standard deviation (SD) from more than three individual experiments; # *p* < 0.05, ## *p* < 0.01, and #### *p* < 0.0001 compared with the control group. * *p* < 0.05, *** *p* < 0.001, and **** *p* < 0.0001 compared with the Dex-stimulated group.

To examine toxicity in zebrafish larvae, we measured survival and heartbeat rates. As shown in Figure 7A, IOE was not toxic at concentrations up to 50 μg/mL. The heartbeat rates of the IOE treated group were not significantly different compared to the control group (Figure 7B). Hence, we used these concentrations to examine the effect of IOE on bone loss in dexamethasone-induced zebrafish larvae. As shown in Figure 7C, dexamethasone inhibited bone mineralization compared to the control group. However, 50 μg/mL IOE significantly increased bone mineralization compared to that seen in the dexamethasone-treated zebrafish larvae. Moreover, we demonstrated the effect of IOE on dexamethasone-induced osteoclast-related gene expression in zebrafish larvae (Figure 8). Dexamethasone induced the expression of mmp9, mmp13, ctsk, foxo1a2, and nfkb2. However, 50 μg/mL IOE significantly inhibited gene expression.

### 2.6. Analysis of DPHC and IPA in IOE

**Figure 9:**
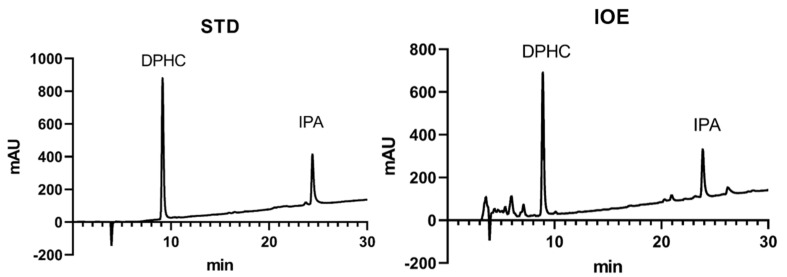
HPLC chromatogram of DPHC and IPA in *I. okamurae*.

We first observed the isolated DPHC and IPA and confirmed the purity of both compounds are over 95%. The retention times of DPHC and IPA were 9.936 and 24.138 min, respectively (Figure 9). The presence of the two compounds in the extract was confirmed by comparing the retention times with those of each standard compound. For the calibration curve, concentration ranges of 0.01–0.08 mg/mL for DPHC and 0.02–0.1 mg/mL for IPA were used. As a result, we found that the IOE comprised 3.13% and 3.42% of DPHC and IPA, respectively.

## 3. Discussion

Osteoclasts facilitate bone resorption during the bone remodeling process. However, excessive activation of osteoclast maturation, differentiation, and activity cause severe bone diseases [33]. Therefore, it is important to identify potential compounds that can inhibit osteoclastogenesis. Our study demonstrated the effect of IOE on osteoclastogenesis and its mechanisms in the RAW 264.7 cells of zebrafish.

Many previous studies have used RANKL to induce the differentiation of RAW 264.7 cells into osteoclasts [34,35]. Therefore, we used RANKL as a stimulator to induce osteoclast differentiation and the generation of osteoclast-related factors in RAW 264.7 cells and investigated the inhibitory activity of IOE. We confirmed that IOE had no cytotoxic effect up to 50 μg/mL, and these concentrations were used in subsequent experiments. TRAP, a histochemical marker of osteoclasts, plays a crucial role in bone resorption [36,37]. RANKL induced an increase in TRAP-positive multinuclear cells, but IOE significantly inhibited the RANKL-induced differentiation of TRAP-positive multinuclear cells in RAW 264.7 cells.

RANKL, a tumor necrosis factor receptor family cytokine, plays a pivotal role in osteoclastogenesis by activating downstream signaling pathways after binding to its receptor RANK [38]; these pathways, including MAPK and NF-κB, are activated following the recruitment of TRAF6 [38]. In osteoclasts, MAPKs, including ERK and JNK, regulate important transcriptional factors via direct or indirect phosphorylation [39]. ERK is involved in the survival, proliferation, formation, disassembly, and differentiation of osteoclasts, and activates c-Fos in response to RANKL [40]. RANKL promotes the stimulation of JNK, which subsequently activates c-Jun [41]. In our study, RANKL treatment activated ERK and JNK, but IOE treatment significantly inhibited the activation of these factors. IOE also inhibited the RANKL-induced expression of c-Fos and c-Jun. Thus, our results suggest that IOE inhibited c-Fos and c-Jun expression by blocking ERK and JNK.

NF-κB is related to osteoclastogenesis as well as MAPKs [42]. TRAF6 recruitment activates the phosphorylation and degradation of IκB. The activated NF-κB p50/p65 heterodimer was ubiquitinated and translocated into the nucleus [43]. The activated MAPK and NF-κB signaling pathways promote the activation of activator protein-1 (AP-1) and NFATc1 [43]. Herein, we observed that IOE inhibits RANKL-induced NF-κB activation and nuclear translocation of p65. The decline in the protein levels of c-Fos and c-Jun following IOE treatment suggests that the inhibition of NF- κB affects the expression of c-Fos and c-Jun. Activator protein-1 (AP-1) comprises c-Jun and c-Fos, which are critical transcription factors for osteoclast formation [43,44]. NFATc1, a member of the NFAT family, is a master regulator that plays a crucial role in RANK-induced osteoclast differentiation [45]. AP-1 promotes the expression of NFATc1 by binding to the NFAT promoter [46]. Upregulated NFATc1 induces the expression of TRAP, CTR, MMP-9, and CTK; hence, these factors promote osteoclastogenesis [47]. In this study, IOE inhibited the RANKL-induced expression of transcriptional factor NFATc1 and osteoclastogenesis-related protein such as CTR, CTK, MMP-9, and TRAP, demonstrating that IOE suppresses the expression of osteoclastogenesis-related proteins by blocking NFATc1. Our findings show that IOE significantly inhibited the RANKL-induced phosphorylation of ERK, JNK, and NF-κB. Therefore, IOE is considered to inhibit osteoclast differentiation via the regulation of AP-1 and NFATc1 by mitigating ERK, JNK, and NF-κB phosphorylation.

Zebrafish (*Danio rerio*) larvae were used as an animal model to confirm the inhibitory effect on osteoporosis. This animal model is widely used owing to its genetic similarity to humans. It has many advantages, such as a short reproductive cycle, small size, and transparent embryos [48,49]. Many researchers have reported a variety of biological activities in zebrafish [50,51,52]. Glucocorticoids (GCs), including dexamethasone and prednisolone, have been widely used to treat diseases related to autoimmunity and inflammation [53,54]. However, the long-term effect of GCs triggers rapid bone resorption by inducing the differentiation and maturation of osteoclasts [55]. Previous studies have reported that dexamethasone treatment induces bone loss as an osteoporosis phenotype in zebrafish larvae [55,56]. Therefore, this study proved the effect of IOE on dexamethasone-induced bone loss in zebrafish larvae. After confirming the non-toxic IOE level, we investigated its inhibitory effect on osteoporosis in the presence of IOE and dexamethasone. Using calcein bone labeling, we demonstrated, for the first time, that IOE has an inhibitory effect on dexamethasone-induced bone loss in zebrafish larvae. Moreover, we verified this mechanism through gene expression analysis. IOE was found to significantly inhibit dexamethasone-induced gene expression, including mmp9, mmp13, foxo1a2, and nfkb. These results indicate that IOE can prevent dexamethasone-induced bone loss in zebrafish larvae by stimulating bone mineralization.

We confirmed that DPHC and IPA are the main compounds in IOE using HPLC analysis. Ihn et al. demonstrated DPHC from *I. okamurae* significantly inhibited osteoclast differentiation by blocking the NF-κB signaling pathway. Moreover, our previous study proved that IPA has an effective inhibitory activity on osteoclastogenesis and leads to an improvement in osteoblastogenesis. In particular, IPA suppressed osteoclast differentiation by downregulating the NF-κB, ERK, and JNK signaling pathways. Our present study has shown that IOE suppresses osteoclastogenesis by inhibiting similar pathways to these compounds. Therefore, this research has shown that DPHC and IPA are the active compounds within IOE.

## 4. Materials and Methods

### 4.1. Extraction of I. okamurae

*I. okamurae* was collected on Jeju Island and washed three times with tap water to remove salt and sand from its surface. The washed samples were dried at room temperature for two weeks. The samples were homogenized using a grinder. Powdered *I. okamurae* (100 g) was subjected to extraction using 70% ethanol (1 L) with continuous agitation at 37 °C, and the solvent was removed under vacuum using a rotary evaporator.

### 4.2. Cell Culture

Murine macrophage RAW 264.7 cells were purchased from the Korean Cell Line Bank (KCLB, Seoul, Republic of Korea). The cells were maintained in Dulbecco’s Modified Eagle’s Medium (Welgene, Gyeongsan, Republic of Korea) containing 10% fetal bovine serum (FBS; Welgene, Republic of Korea) and 1% antibiotic–antimycotic (Gibco BRL, San Diego, CA, USA) at 37 °C in a humidified incubator with 5% CO_2_.

### 4.3. MTT Assay

We seeded 2 × 10^4^ RAW 264.7 cells/mL in a 96-well plate. After 24 h, 12.5, 25, 50, and 100 μg/mL of IOE were added for 5 days. MTT assay was performed according to the method reported by Cho et al. [32]. The absorbance was read at 540 nm using a SpectraMax M2/M2e spectrophotometer (Molecular Devices, San Jose, CA, USA).

### 4.4. TRAP Staining

We seeded 2 × 10^4^ RAW 264.7 cells/mL in a 24-well plate. After 24 h, the cells were pre-treated with 25, 50, and 100 μg/mL of IOE for 2 h and then treated with 100 ng/mL RANKL (Sigma-Aldrich, St. Louis, MO, USA) for 5 days. The medium was replaced every 2 days. TRAP activity was measured according to the method reported by Cho et al. [32]. TRAP-positive cells were observed and counted under a light microscope (Carl Zeiss, Oberkochen, Germany). The number of TRAP-positive cells was calculated as a ratio compared to the total number of cells.

### 4.5. Western Blotting Analysis

Protein levels were analyzed according to the method reported by Cho et al. [32]. The membranes were incubated overnight at 4 °C with the following primary antibodies: anti-TRAP, anti-MMP-9, anti-CTR, anti-CTK (Abcam, Cambridge, MA, USA), anti-NFATc1 (BD Pharmingen^TM^, San Diego, CA, USA), anti-c-Fos, anti-c-Jun, anti-phospho-extracellular signal-regulated kinase (ERK), anti-ERK, anti-phospho-c-Jun N-terminal kinase (JNK), anti-JNK, anti-phospho-IκB, anti-phospho-p65 (Cell Signaling Technology, Danvers, MA, USA) anti-phospho-p50, and anti-β-actin (Santa Cruz Biotechnology, Dallas, TX, USA). Membranes washed with TBST (20 mM Tris, 137 mM sodium chloride, 0.1% Tween-20; Sigma-Aldrich, USA) were incubated for 2 h at room temperature with the following secondary antibodies: anti-rabbit IgG, HRP-linked antibody, anti-mouse IgG, and HRP-linked antibody (Cell Signaling Technology, Danvers, MA, USA). The membranes were then washed three times with TBST. Protein bands were observed using a SuperSignal West Femto Trial kit (Thermo Fisher Scientific, Waltham, MA, USA) using the Fusion FX Chemiluminescence system (Vilber Lourmat, Collégien, France).

### 4.6. Immunofluorescence (IF) Staining

We seeded 5 × 10^4^ cells/mL RAW 264.7 cells/mL in a confocal chamber. The cells were pre-treated with 100 μg/mL IOE for 2 h and then treated with 100 ng/mL RANKL for 5 min. IF staining was performed according to the method described by Cho et al. [32]. The cells were incubated overnight at 4 °C with the following primary antibodies: anti-p65. After being washed another three times with PBS, the cells were incubated for 1.5 h at room temperature with the following the secondary antibodies: Alexa fluor488-labeled goat anti-rabbit IgG (H + L) cross-adsorbed secondary antibody (1:800; Thermo Fisher Scientific, USA). Washed cells with PBS are treated with 40 μg/mL Hoechst 33,342 to stain the nucleus for 10 min at room temperature. Washed cells were mounted with ProLongTM Gold antifade mountant (Thermo Fisher Scientific, USA). Fluorescence signals were detected using a LSM 700 Zeiss confocal laser scanning microscope (Carl Zeiss, Germany).

### 4.7. Maintenance of Zebrafish and Survival Rate Measurement

Zebrafish (*D. rerio*) were purchased from a local pet market and maintained in a circulating system under the following conditions: 28.5 ± 1 °C with a 14/10 h light/dark cycle. The zebrafish were fed twice daily. One female and two males were randomly selected from the breeding cages to obtain embryos. The embryos were collected, placed in 1 mg/mL methylene blue solution for 1 h, and transferred to fresh embryo media (600 mg/mL sea salt in distilled water). Fertilized embryos were maintained at 28.5 °C for 72 h until the larvae hatched. Zebrafish larvae feeding was initiated 5 days post-fertilization (dpf). Experiments were initiated at 10 dpf. The survival rate was measured at 11–16 dpf in the presence of 5, 10, 20, 50, and 100 μg/mL of IOE. The heartbeat rate was measured for 1 min under the microscope. The experiments were approved by the Animal Care and Use Committee of the Korea Basic Science Institute, Daejeon, Republic of Korea (KBSI-IACUC-23-25).

### 4.8. Calcein Staining

Calcein is a fluorescent marker used to detect calcium [57]. Zebrafish larvae (10 dpf) were placed in a 6-well plate in each well and treated with 50 μg/mL of IOE and 25 μM of dexamethasone for 3 days. Calcein solution (0.2%) was prepared by dissolving 0.1 g of calcein powder (Sigma-Aldrich, USA) and 50 mL of distilled water. The larvae were washed with distilled water, and 0.2% calcein solution was added for 15 min at room temperature. Stained larvae were fixed with 4% PFA and then rinsed with distilled water for 30 min to remove unbound and excess calcein. The larvae were visualized under a LSM 700 Zeiss confocal laser scanning microscope.

### 4.9. Real-Time Quantitative Reverse Transcription Polymerase Chain Reaction (RT-qPCR)

Total mRNA was isolated from zebrafish larvae using TRIzol according to the manufacturer’s instructions (Invitrogen, Carlsbad, CA, USA). cDNA synthesis was performed using the following reagents: RNase-free DNase I (Promega, Fitchburg, WI, USA), SUPERase-in (Ambion, Austin, TX, USA), EDTA (Promega, USA), dNTP (Invitrogen, USA), random primers (Invitrogen, USA), and reverse transcriptase (Promega, USA). Synthesized cDNA was then used for PCR amplification with SYBR Green (Applied Biosystems, San Francisco, CA, USA). Relative mRNA expression was calculated by relative quantification using comparative threshold cycle (CT) values based on β-actin levels according to the manufacturer’s instructions (Applied Biosystems). Primers used for real-time qPCR are described in Table 1.

### 4.10. High Performance Liquid Chromatography (HPLC) Analysis

In our previous study, DPHC was identified in the ethyl acetate fraction by HPLC and LC/mass spectrometry (MS) analysis and showed antioxidant activity [58]. As mentioned in our previous study, the column was eluted in gradient mode with a mobile phase solvent system containing acetonitrile and water (acetonitrile-water [0–50 min: 5:95–95:5 *v*/*v*; 50–60 min: ~100:0 *v*/*v*]) with a flow rate of 0.3 mL/min, while motoring the absorbance at 230 nm.

### 4.11. Statistical Analysis

The data are expressed as mean ± standard deviation (SD) and were analyzed by one-way ANOVA with Tukey’s post hoc test. Data were considered statistically significant at *p* < 0.05. All statistical tests were performed using GraphPad Prism software (version 8.0; GraphPad Software, San Diego, CA, USA).

## 5. Conclusions

This study demonstrated the anti-osteoporosis effects of IOE in vitro and in vivo. IOE significantly inhibited RANKL-induced osteoclast differentiation by inhibiting NFATc1 and AP-1 via the blockade of ERK, JNK, and NF-κB signaling pathways. Dexamethasone-induced bone loss in zebrafish larvae was also evaluated, revealing that IOE significantly inhibited dexamethasone-induced bone loss. Taken together, our study indicates that IOE has a potential use in the treatment of bone diseases, including osteoporosis.

## Figures and Tables

**Table 1:** List of primer sequences used for RT-qPCR.

Gene	Forward Primer	Reverse Primer
mmp9	tcggcctaccaagcgactt	tcatgtgaatcaatgggcactc
mmp13	agaccaggacacactcgcagag	tcgggccgcatctcttcact
nfkb2	acaagacgcaaggagcccag	aactgtctcttgcacaaagggc
foxo1a2	cgcatccccagcaacagcat	aatgtggacctcggctgcct
β-actin	ccatccttcttgggtatgga	acaggtccttacggatgtc

## Data Availability

The data presented in this study are available on request from the corresponding author.

## References

[1] Meguid E.A., Ke Y., Ji J., El-Hashash A.H.K. (2018). Stem cells applications in bone and tooth repair and regeneration: New insight, tools, and hops. J. Cell. Physiol..

[2] Kim J.H., Kim N. (2020). Bone cell communication factors provide a new therapeutic strategy for osteoporosis. Chonnam Med. J..

[3] Lee K.Y., Kim J.H., Kim E.Y., Yeom M., Jung H.S., Sohn Y. (2019). Water extract of *Cnidii Rhizoma* suppresses RANKL-induced osteoclastogenisis in RAW 264.7 cell by inhibiting NFATc1/c-Fos signaling and prevents ovariectomized bone loss in SD-rat. BMC Complement. Altern. Med..

[4] Katsimbri P. (2017). The biology of normal bone remodeling. Eur. J. Cancer Care.

[5] Phetfong J., Sanvoranart T., Nartprayut K., Nimsanor N., Seenprachawong K., Prachayasittikul V., Supokawej A. (2016). Osteoporosis: The current status of mesenchymal stem cell-based therapy. Cell. Mol. Biol. Lett..

[6] Yin Z., Zhu W., Wu Q., Zhang Q., Guo S., Liu T., Li S., Peng D. (2019). Glycyrrhizic acid suppresses osteoclast differentiation and postmenopausal osteoporosis by modulating the NF-κB, ERK, and JNK signaling pathways. Eur. J. Pharmacol..

[7] Liu X., Chin J.F., Qu X., Bi H., Liu Y., Yu Z., Zhai Z., Qin A., Zhang B., Dai M. (2017). The beneficial effect of praeruptorin C on osteoporotic bone in ovariectomized mice via suppression of osteoclast formation and bone resorption. Front. Pharmacol..

[8] Tokegahara N., Kim H., Choi Y. (2022). RANKL biology. Bone.

[9] Kim M., Kim H.S., Kim J.H., Kim E.Y., Lee B., Lee S.Y., Jun J.Y., Kim M.B., Sohn Y., Jung H.S. (2020). Chaenomelis fructus inhibits osteoclast differentiation by suppressing NFATc1 expression and prevents ovariectomy-induced osteoporosis. BMC Complement. Med. Ther..

[10] Xu H., Chen F., Liu T., Xu J., Li J., Jiang L., Wang X., Sheng J. (2020). Ellagic acid blocks RANKL-RANK interaction and suppresses RANKL-induced osteoclastogenesis by inhibiting RANK signaling pathways. Chem. Biol. Interact..

[11] Zhu W., Yin Z., Zhang Q., Guo S., Shen Y., Liu T., Liu T., Liu B., Wan L., Li S. (2019). Proanthocyanidins inhibits osteoclast formation and function by inhibiting the NF-κB and JNK signaling pathways during osteoporosis treatment. Biochem. Biophys. Res. Commun..

[12] Thu H.E., Hussain Z., Mohamed I.N., Shuid A.N. (2019). *Eurycoma longifolia*, a promising suppressor of RANKL-induced differentiation and activation of osteoclasts: An in vitro mechanistic evaluation. J. Ayurveda Integr. Med..

[13] Dömötör Z.R., Vörhendi N., Hanák L., Hegyi P., Kiss S., Csiki E., Szakó L., Párniczky A., Erőss B. (2020). Oral treatment with bisphosphonates of osteoporosis does not increase the risk of severe gastrointestinal side effects: A metal-analysis of randomized controlled trials. Front. Endocrinol..

[14] Imam B., Aziz K., Khan M., Zubair T., Iqbal A. (2019). Role of bisphosphonate in postmenopausal women with osteoporosis prevent future fractures: A literature review. Cureus.

[15] Kennell K.A., Drake M.T. (2009). Adverse effects of bisphosphonates: Implications for osteoporosis management. Mayo Clin. Proc..

[16] Elkordy A.A., Haj-Ahmad R.R., Awaad A.S., Zaki R.M. (2021). An overview on natural product drug formulations from conventional medicines to nanomedicines: Past, present and future. J. Drug Deliv. Sci. Technol..

[17] Lam K.S. (2007). New aspects of natural products in drug discovery. Trends Microbiol..

[18] Yang H.W., Son M., Choi J., Oh S., Jeon Y.J., Byun K., Ryu B. (2019). Effect of isophloroglucin A, a component of *Ishige okamurae*, on glucose homeostasis in the pancreas and muscle of high fat diet-red mice. Mar. Drugs.

[19] Kiuru P., D’Auria M.V., Muller C.D., Tammela P., Vuorela H., Yli-Kauhaluoma J. (2014). Exploring marine resources for bioactive compounds. Planta Medica.

[20] Fernando K.H.N., Tang H.Y., Jiang Y., Jeon Y.J., Ryu B. (2019). *Ishige okamurae* extract and its constituent ishophloroglucin A attenuated in vitro and in vivo high glucose-induced angiogenesis. Int. J. Mol. Sci..

[21] Wang L., Oh J.Y., Jayawardena T.U., Jeon Y.J., Ryu B. (2020). Anti-inflammatory and anti-melanogenesis activities of sulfated polysaccharides isolated from *Hizikia fusiforme*: Short communication. Int. J. Biol. Macromol..

[22] Jun J.Y., Jung M.J., Jeong I.M., Yamazaki K., Kawai Y., Kim B.M. (2018). Antimicrobial and antibiofilm activities of sulfated polysaccharides from marine algae against dental plaque bacteria. Mar. Drugs.

[23] Sanjeewa K.K.A., Lee W.W., Kim J.I., Jeon Y.J. (2017). Exploiting biological activities of brown seaweed *Ishige okamurae* Yendo for potential industrial applications: A review. J. Appl. Phycol..

[24] Lee K.M., Boo G.H., Riosmena-Rodriguez R., Boo S.M. (2009). Classification of the genus Ishige (Ishigeales, Pyaeophyceae) in the north pacific ocean with recognition of *Ishige foliacea* based on plastid rbcl and mitochondrial cox3 gene sequences(1). J. Phycol..

[25] Min K.H., Kim H.J., Jeon Y.J., Han J.S. (2011). *Ishige okamurae* ameliorates hyperglycemia and insulin resistance in C57BL/KsJ-db/db-mice. Diabetes Res. Clin. Pract..

[26] Kwon O.Y., Lee S.H. (2023). *Ishige okamurae* attenuates neuroinflammation and cognitive deficits in mice intracerebroventricularly injected with LPS via regulating TLR-4/MyD88-dependent pathways. Antioxidants.

[27] Ahn M.J., Yoon K.D., Kim C.Y., Kim J.H., Shin C.G., Kim J. (2006). Inhibitory activity on HIV-1 reverse transcriptase and integrase of a carmalol derivative from a brown Alga, *Ishige okamurae*. Phytother. Res..

[28] Das S.K., Ren R., Hashimoto T., Kanazawa K. (2010). Fucoxanthin induces apoptosis in osteoclast-like cells differentiated from RAW 264.7 cells. J. Agric. Food Chem..

[29] Rahim A.H., Setiawan B., Dewi F.R.P., Noor Z. (2015). Regulation by phloroglucinol of Nrf2/Maf-mediated expression of antioxidant enzymes and inhibition of osteoclastogenesis via the RANKL/RANK signaling pathway: In silico study. Acta Inform. Med..

[30] Ihn H.J., Kim J.A., Cho H.S., Shin H.I., Kim G.Y., Choi Y.H., Jeon Y.J., Park E.K. (2017). Diphlorethohydroxycarmalol from *Ishige okamurae* suppresses osteoclast differentiation by downregulating the NF-κB signaling pathway. Int. J. Mol. Sci..

[31] Ryu B., Jiang Y., Kim H.S., Hyun J.M., Lim S.B., Li Y., Jeon Y.J. (2018). Ishophloroglucin A, a novel phlorotannin for standardizing the anti-α-glucosidase activity of *Ishige okamurae*. Mar. Drugs.

[32] Cho S.H., Kim H.S., Jung H.Y., Park J.I., Jang Y.J., Ahn J., Kim K.N. (2023). Effect of Ishophloroglucin A isolated from *Ishige okamurae* on in vitro osteoclastogenesis and osteoblastogenesis. Mar. Drugs.

[33] Singh A., Dutta M.K., Jennane R., Lespessailles E. (2017). Classification of the trabecular bone structure of osteoporotic patients using machine vision. Comput. Biol. Med..

[34] Jeong S., Lee S., Kim K., Lee Y., Lee J., Oh S., Choi J.W., Kim S.W., Hwang K.C., Lim S. (2020). Isoliquiritigenin derivatives inhibits RANKL-induced osteoclastogenesis by regulating p38 and NF-κB activation in RAW 264.7 cells. Molecules.

[35] Orecchini E., Mondanelli G., Orabona C., Volpi C., Adorisio S., Calvitti M., Thuy T.T., Delfino D.V., Belladonna M.L. (2021). *Artocarpus tonkinensis* extrct inhibits LPS-triggered inflammation markers and suppresses RANKL-induced osteoclastogenesis in RAW 264.7. Front. Pharmacol..

[36] Harada K., Itoh J., Kawazoe Y., Miyazaki S., Doi K., Kubo T., Akagawa Y., Shiba T. (2013). Polyphosphate-mediated inhibition of Tartrate-resistant acid phosphatase and suppression of bone resorption of osteoclasts. PLoS ONE.

[37] Kang I.S., Kim C. (2019). Taurine chloramine inhibits osteoclastic differentiation and osteoclast marker expression in RAW 264.7 cells. Adv. Exp. Med. Biol..

[38] Park J.H., Lee N.K., Lee S.Y. (2017). Current understanding of RANKL signaling in osteoclast differentiation and maturation. Mol. Cells.

[39] Yan L., Lu L., Hu R., Shetti D., Wei K. (2019). Piceatannol attenuates RANKL-induced osteoclast differentiation and bone resorption by suppressing MAPK, NF-κB and AKT signaling pathways and promotes caspase-3-mediated apoptosis of mature osteoclasts. R. Soc. Open Sci..

[40] Lee K., Seo I., Choi M.H., Jeon D. (2018). Roles of mitogen-activated protein kinases in osteoclast biology. Int. J. Mol. Sci..

[41] Tankak S., Nakamura K., Takahasi N., Su T. (2005). Role of RANKL in physiological and pathological bone resorption and therapeutics targeting the RANKL-RANK signaling system. Immunol. Rev..

[42] Wu M., Wang Y., Deng L., Chen W., Li Y.P. (2012). TRAP family member-associated NF-κB activator (TANK) indued by RANKL negatively regulates osteoclast survival and function. Int. J. Biol. Sci..

[43] Huang X.L., Huang L.Y., Cheng Y.T., Li F., Zhou Q., Wu C., Shi Q.H., Guan Z.Z., Liao J., Hong W. (2019). Zoledronic acid inhibits osteoclast differentiation and function through the regulation of NF-κB and JNK signaling pathway. Int. J. Mol. Sci..

[44] Jang H.Y., Lee H.S., Noh E.M., Kim J.M., You Y.O., Lee G., Koo J.H., Lim H., Ko S., Kim J.S. (2021). Aqueous extract of *Chrysanthemum morifolium* Ramat. inhibit RANKL-induced osteoclast differentiation by suppressing the c-fos/NFATc1 pathway. Arch. Oral Biol..

[45] Kim J.H., Kim N. (2014). Regulation of NFATc1 in osteoclast differentiation. J. Bone Metab..

[46] Chiou W.F., Huang T.L., Liu Y.W. (2014). (+)-Vitisin A inhibits osteoclast differentiation by preventing TRAF6 ubiquitination and TRAF6-TAK1 formation to suppress NFATc1 activation. PLoS ONE.

[47] Yang J., Kim J.H., Kim M., Ryu G.H., Moon J.H., Lee H.I., Jung H.S., Sohn Y. (2020). *Gentianae macrophyllae* Radix water extract inhibits RANKL-induced osteoclastogenesis and osteoclast specific genes. Korean J. Acupunct..

[48] Dai Y.J., Jia Y.F., Chen N., Bian W.P., Li Q.K., Ma Y.B., Chen Y.L., Pei D.S. (2014). Zebrafish as a model system to study toxicology. Environ. Toxicol. Chem..

[49] Veldman M.B., Lin S. (2008). Zebrafish as a developmental model organism for pediatric research. Pediatr. Res..

[50] Fehrmann-Cartes K., Coronado M., Hernández A.J., Allende M.L., Feijoo C.G. (2019). Anti-inflammatory effects of aloe vera on soy meal-induced intestinal inflammation in zebrafish. Fish Shellfish Immunol..

[51] Nguyen T.H., Le H.D., Kim T.N.T., The H.P., Nguyen T.M., Cornet V., Lambert J., Kestemont P. (2020). Anti-inflammatory and antioxidant properties of the ethanol extract of *Clerodendrum cyrtophyllum* Turcz in copper sulfate-induced inflammation in zebrafish. Antioxidants.

[52] Chen Y.M., Su W.C., Li C., Shi Y., Chen Q.X., Zheng J., Tang D.L., Wang Q. (2019). Anti-melanogenesis of novel kojic acid derivatives in B16F10 cells and zebrafish. Int. J. Biol. Macromol..

[53] Ronchetti S., Ayroldi E., Ricci E., Gentili M., Migliorati G., Riccardi C. (2021). A glance at the use of glucocorticoids in rare inflammatory and autoimmune diseases: Still an indispensable pharmacological tool?. Front. Immunol..

[54] Bordag N., Klie S., Jürchott K., Vierheller J., Schiewe H., Albrecht V., Tonn J.C., Schwart C., Schichor C., Selbig J. (2015). Glucocorticoid (dexamethasone)-induced metabolome changes in healthy males suggest prediction of response and side effect. Sci. Rep..

[55] Luo S.Y., Yang Y., Chen J., Zhong Z., Huang H., Zhang J., Cui L. (2016). Tanshinol stimulates bone formation and attenuates dexamethasone-induced inhibition of osteogenesis in larvae zebrafish. J. Orthop. Transl..

[56] Luo S.Y., Chen J.F., Zhong Z.G., Lv X.H., Yang Y.J., Zhang J.J., Cui L. (2016). Salvianolic acid B stimulates osteogenesis in dexamethasone-treated zebrafish larvae. Acta Pharmacol. Sin..

[57] Chen J.R., Lai Y.H., Tsai J.J., Hsiao C.D. (2017). Live fluorescent staining platform for drug-screening and mechanism-analysis in zebrafish for bone mineralization. Molecules.

[58] Kim H.S., Wang L., Jayawardena T.U., Kim E.A., Heo S.J., Fernando I.P.S., Lee J.H., Jeon Y.J. (2020). High-performance centrifugal partition chromatography (HPCPC) for efficient isolation of diphlorethohydroxycarmalol (DPHC) and screening of its antioxidant activity in a zebrafish model. Process Biochem..

